# Plant–Plant Communication: Is There a Role for Volatile Damage-Associated Molecular Patterns?

**DOI:** 10.3389/fpls.2020.583275

**Published:** 2020-10-15

**Authors:** Anja K. Meents, Axel Mithöfer

**Affiliations:** Research Group Plant Defense Physiology, Max Planck Institute for Chemical Ecology, Jena, Germany

**Keywords:** DAMP, defense, plant–plant communication, signaling, volatiles, wounding

## Abstract

Damage-associated molecular patterns (DAMPs) are an ancient form of tissue-derived danger or alarm signals that initiate cellular signaling cascades, which often initiate defined defense responses. A DAMP can be any molecule that is usually not exposed to cells such as cell wall components, peptides, nucleic acid fragments, eATP and other compounds. DAMPs might be revealed upon tissue damage or during attack. Typically, DAMPs are derived from the injured organism. Almost all eukaryotes can generate and respond to DAMPs, including plants. Besides the molecules mentioned, certain volatile organic compounds (VOCs) can be considered as DAMPs. Due to their chemical nature, VOCs are supposed to act not only locally and systemically in the same plant but also between plants. Here, we focus on damage-induced volatiles (DIVs) that might be regarded as DAMPs; we will review their origin, chemical nature, physiochemical properties, biological relevance and putative function in plant–plant communications. Moreover, we discuss the possibility to use such airborne DAMPs as eco-friendly compounds to stimulate natural defenses in agriculture in order to avoid pesticides.

## Introduction

As other eukaryotic organisms, plants are able to perceive typical, endogenous cell molecules or fragments thereof, when these are released at increased concentrations into the extracellular space. This occurs during cellular stress or mechanical damage upon herbivore and pathogen attack. Subsequently, the endogenous compounds contribute to activate local and systemic defense-related responses or the plant innate immunity (Howe and Jander, 2008; Boller and Felix, 2009). The whole dynamic immunity response is induced by the recognition of specific insect-derived [herbivore-associated molecular patters (HAMPs) (Mithöfer and Boland, 2008)] or pathogen-derived [pathogen-associated molecular patters (PAMPs) (Ausubel, 2005)] signals, and signals from the injured plant cells. These latter signaling molecules function as danger signals, stress signals, (endogenous) elicitors, alarmins, or damage-associated molecular patterns (DAMPs). Although various synonyms exist for the aforementioned molecules, the term DAMP is to our knowledge the most prominent example and will be further referred to in this review. With the increasing acceptance of the “damaged-self recognition” concept (Heil, 2009) for plants, the number of DAMPs, their putative reception and signaling and the corresponding literature continuously increased. Thus, here we avoid providing another collection of DAMPs and refer to recent reviews that give comprehensive overviews (Boller and Felix, 2009; Heil and Land, 2014; Gust et al., 2017; Quintana-Rodriguez et al., 2018; Hou et al., 2019; Ferrusquía-Jiménez et al., 2020). Nevertheless, some typical examples must be mentioned such as peptides, cell wall components, nucleic acid fragments, and extracellular ATP (eATP). However, a new putative class of DAMPs that would be unique for plants (Heil and Land, 2014) will be addressed in the following: volatile DAMPs.

In recent years, plant-derived volatile organic compounds (VOCs) gained much attention as cues in plant–plant communication. However, the concept of VOCs released by attacked plants transmitting information to warn neighboring individuals is far from posing as a novelty, being described almost 40 years ago in various caterpillar-infested tree species (Baldwin and Schultz, 1983; Rhoades, 1983). Criticism regarding the lack of true replication and artificial experimental conditions (Fowler and Lawton, 1985) resulted in the rejection of this popular phenomenon known as “talking trees.” It took almost 20 years to revisit and revive the concept of plant–plant communicating via volatile cues by intensely searching for evidence of VOC-induced plant protection against herbivory (Heil and Karban, 2010; Karban et al., 2014). This review focuses specifically on wounding-/damage-induced plant volatiles that fulfill the criteria of DAMPs in *stricto sensu*. We highlight their chemical nature and their ability to induce defense responses in neighboring plants and critically examine their putative role in the field.

## A Short Survey of Plant Volatiles

**FIGURE 1:**
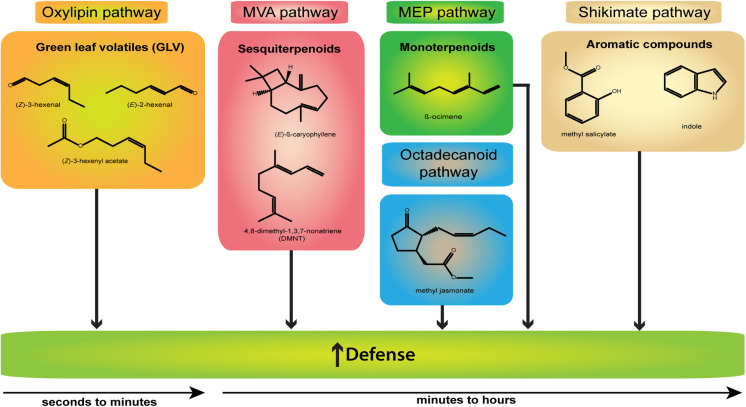
Representative damage-induced volatiles (DIVs). Shown are structures and the biosynthetic origin (indicated by different colors) of the main DIVs involved in defense response induction (↑) and released after wounding or mechanical damage only. Compounds of the oxylipin pathway appear early within seconds to minutes, other compounds later within minutes to hours.

A plethora of studies is available highlighting the versatility of VOCs and in particular of herbivory-induced plant volatiles (HIPVs). Apart from activating direct and indirect plant defenses against herbivores, HIPVs are also known to mediate a diverse array of interactions between plants and insects (Turlings et al., 1990; De Moraes et al., 1998, 2001; Hoballah and Turlings, 2001). In numerous plant species HIPVs are involved in repelling herbivores, attracting their predators of a higher trophic level as well as upregulating and priming defense responses (Kessler and Baldwin, 2002; Arimura et al., 2004; Engelberth et al., 2004; Kessler et al., 2006; Arimura and Pearse, 2017). Although plants release distinct volatile bouquets with differing compositions and concentrations depending on the given stimulus, e.g., herbivory, mechanical wounding, or touch (Mithöfer et al., 2005; Bricchi et al., 2010; Meents et al., 2019), many taxa share common constituents (McCormick et al., 2012). The most well-known representatives described within the past decades are terpenoids, phenylpropanoids as well as fatty acid and amino acid derivatives (Figure 1) (Dudareva et al., 2004, 2006).

Among the most ubiquitous VOCs emitted after mechanical damage, herbivory, or microbial infection are green leaf volatiles (GLVs, for a full review see, Ameye et al., 2018) named after their typical odor of freshly cut green leaves. GLVs are C_6_ alcohols, aldehydes, and esters such as (*Z*)-3-hexenal, (*E*)-2-hexenal, (*Z*)-3-hexen-1-ol, and (*Z*)-3-hexen-1-yl acetate generated via oxidation of fatty acids such as linoleic and α-linolenic acid within the oxylipin pathway (for example, see, Matsui, 2006).

Considering the largest class of plant secondary metabolites, terpenes provide a wide array of volatile compounds which are subdivided depending on the number of C_5_ units (Dudareva et al., 2004; McCormick et al., 2012). The main representatives of this family are hemiterpenes (C_5_; e.g., isoprene), monoterpenes [C_10_; e.g., linalool, *(E)-*β-ocimene], sesquiterpenes [C_15_; *(E)-*β-caryophyllene, *(E,E)-*α-farnesene, α-humulene], and homoterpenes displaying irregular structures such as *(E)-*4,8-dimethyl-1,3,7-nonatriene (DMNT; C_11_) and (*E,E*)-4,8,12-trimethyltrideca-1,3,7,11-tetraene (TMTT; C_16_) (Boland et al., 1992; Maffei et al., 2011; McCormick et al., 2012). The formation of the abovementioned terpenes from the basic C_5_ building blocks occurs via two compartmentalized pathways: the cytosol-localized mevalonate pathway (MVA) and the methylerythritol phosphate (MEP) pathway localized in the plastids (Dudareva et al., 2004). Both pathways are strictly enzymatically regulated by a large family of terpene synthases (Dudareva et al., 2013).

Another structurally diverse category of VOCs are the shikimate pathway-derived phenylpropanoids and benzenoids, originating from the amino acid phenylalanine. Sharing a single or multiple benzene rings, these two classes undergo miscellaneous modifications such as acetylation, hydroxylation or methylation, thereby creating a variety of side chains and resulting compounds (Dudareva et al., 2004, 2006). Being often specific to certain plant species and genera, methyl salicylate (MeSA), benzaldehyde, chavicol, eugenol, phenylethanol, and benzylalcohol are typical compounds of this category which can be found in numerous volatile bouquets (Dudareva et al., 2006; Arimura and Pearse, 2017). By performing radioactive labeling studies, several other amino acid-derived VOCs ranging from, e.g., isothiocyanates, sulfides, nitriles, oximes, and amines have been discovered over the years (Dudareva et al., 2006; McCormick et al., 2012). One of the key volatiles released after herbivore damage is indole, which is biosynthesized via anthranilate as an intermediate product in the tryptophan pathway (Paré and Tumlinson, 1996; Frey et al., 2000).

In the context of plant volatiles and their effects on atmospheric chemistry, short-chain oxygenated volatiles (oxVOCs) such as formic and acetic acids, formaldehyde, acetone, methanol, and ethanol, have gained increasing importance in research respective to climate change and contribution to formation of aerosol particles and ozone (Seco et al., 2007).

## Mechanical Damage-Induced VOCs

**FIGURE 2:**
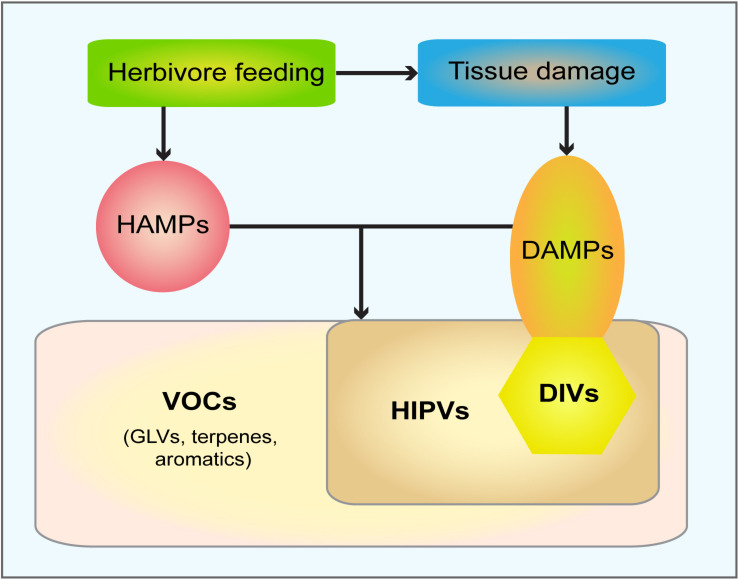
Scheme of the relationship between groups of volatile compounds induced upon herbivore feeding and tissue damage. Herbivore feeding provides chemical signals, HAMPs (herbivory-associated molecular patters), and causes tissue damage, which in turn generates DAMPs (damage-associated molecular patters). The combination of HAMPs and DAMPs induce the emission of HIPVs (herbivory-induced plant volatiles), which all belong to the huge group of VOCs (volatile organic compounds) that includes GLV (green leaf volatiles), terpenes and aromatic compounds. DAMPs are also generated by tissue damage/wounding alone. DIVs (damage-induced volatiles) represent a sub-group of DAMPs, due to their volatile character; all DIVs belong to HIPVs. For simplicity damage-induced electrical signals are neglected.

**FIGURE 3:**
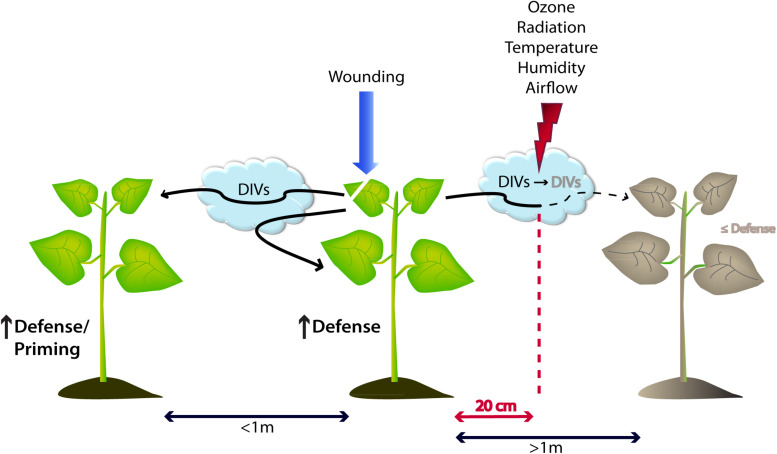
Model of damage-induced volatile (DIV) emissions that trigger intra- and interspecific defense responses in plants. Upon wounding events without contribution from other organisms, plants release specific DIVs possessing the ability to upregulate molecular and chemical defense mechanisms within the same individual as well as in neighboring plants up to a distance of 1 m. The signal intensity and distance of DIVs is highly dependent on environmental factors such as tropospheric reagents (ozone), temperature, radiation as well as the direction of airflow. All of the aforementioned conditions can drastically reduce the effectiveness of DIV signaling by lowering it to a ∼20 cm radius.

While studies investigating HIPVs became increasingly popular over time, volatiles solely induced by and emitted after mechanical damage [from now on *damage-induced volatiles* (DIVs)] without any contribution of other organisms, were predominantly shortly mentioned or being considered as not representative for natural processes. In more recent years, VOCs received increasing attention as a DAMP-related cue whilst serving as reliable responses upon damage in various plant tissues. In Figure 2 different key players involved in volatile induction and their relationship among each other are depicted. Particular studies by Quintana-Rodriguez et al. (2018) placed volatiles emitted upon wounding-induced tissue damage in the absence of elicitors in an entirely new context. They pointed out that such DIVs are synthesized upon cell disruption and possess the ability to trigger systemic responses and herbivore resistance, therefore functioning as a DAMP in plants (Figure 3) (Heil, 2009; Duran-Flores and Heil, 2016). However, it is also conceivable that DIVs are generated downstream of classical DAMPs such as oligosaccharines or peptides and therefore should be seen as second messengers rather that the initial signals. Most likely, DIVs are synthetized *de novo* after damage. However, here we must discriminate two situations. First, synthesis is initiated within seconds upon tissue damage by constitutively present enzymes as in case of GLVs. Second, synthesis is induced only upon damage perception within hours as for example in case of phenolic compounds and many terpenes. In any case the release of all these volatile compounds can be considered as early and late damage-induced responses, respectively; in contrast to classic DAMPs which are not synthesized upon damage. Only some stored terpenes are released immediately upon disruption of tissue containing pre-existing secretory structures.

Considering that damaging of a plant without the introduction of foreign molecular patterns (e.g., insects) completely omits evolutionary factors such as arms race (Heil, 2009), investigation of the underlying mechanisms will improve the understanding of “ancient” plant defense responses. Thus, we next would like to take a closer look which volatiles are actually released solely upon mechanical damage without associated herbivore feeding or other stress factors in order to identify DIVs, which might serve as potential ancient DAMPs.

One of the most well-known DIVs is the characteristic smell of freshly cut grass, mainly caused by the emission of GLVs. Karl et al. (2001) identified predominantly C_6_ compounds including (*Z*)-3-hexenal, (*E*)-2-hexenal, hexenol, hexanal, and acetaldehyde to be emitted within minutes after lawn mowing and lasting for several hours in the field, therefore causing this distinct bouquet. The rapid emission reported in this field study confirmed previous findings in aspen (*Populus tremuloides*), beech (*Fagus sylvatica*), and clover (*Trifolium repens*), where cutting of leaves with scissors elicited a release of (*Z*)-3-hexenal within 1–2 s paving the way for the release of the aforementioned compounds plus hexenyl acetates (Fall et al., 1999). The sensitivity of such measurements was immensely improved by new measuring techniques, such as proton−transfer−reaction mass spectrometry (PTR-MS), enabling monitoring of the release of selected VOCs simultaneous and on-line in the laboratory or in the field.

In addition to the aforesaid rapidly emitted GLVs, mechanical wounding has been shown to generate a variety of DIVs in many different species ranging from common agricultural crops (tomato, *Solanum lycopersicum*; potato, *Solanum tuberosum*; lima bean, *Phaseolus lunatus*), model organisms (*Arabidopsis thaliana;* common liverwort, *Marchantia polymorpha*), herbs, shrubs, and grasses (sagebrush, *Artemisia tridentata*; common reed, *Phragmites australis*; *Plantago lanceolata*) to even trees (aspen, *Populus tremula*; beech, *Fagus sylvatica*; poplar, *Populus nigra*). Although the emission of such DIVs occurs in a species and/or cultivar dependent manner, similar constituents are found in the emitted bouquets.

Jackson and Campbell (1976) observed the release of the plant hormone ethylene after excision of petiole segments from tomato plants. In the following years the list of known DIVs became increasingly refined, adding β-caryophyllene, (*E*)-β-farnesene, germacrene D, and β-bisabolene discovered in potato and common broad bean (*Vicia faba*), to the mix (Agelopoulos et al., 1999). Headspace analyses in *A. thaliana* revealed, apart from GLVs, an increased emission of various terpenoids (β-ionone, β-cyclocitral), sulfides (dimethyl disulfide, dimethyl trisulfide), alcohols (3-pentanol, 1-penten-3-ol, 2-ethyl-1-hexanol), and ketones (3-pentanone, 1-penten-3-one) after rubbing of the leaf midrip with carborundum powder (Van Poecke et al., 2001). Using a more common wounding approach by punching holes into lima bean leaves, Arimura et al. (2000) paved the way for extensive VOC studies using this species by demonstrating the upregulated release of, e.g., DMNT, MeSA, α-pinene (in addition to previously mentioned compounds). From the 2000s onwards, more and more DIVs comprising methyl jasmonate (MeJA) in sagebrush (Preston et al., 2001), linalool and linalool oxide in damaged wheat (*Triticum aestivum*) (Piesik et al., 2006), acetaldehyde, methanol, isoprene, and additional C_6_ compounds in common reed (Loreto et al., 2006), the essential oils pulgeone and menthone in the medicinal plant *Minthostachys mollis* (Banchio et al., 2005), as well as C_8_ VOCs in the model liverwort species *Marchantia polymorpha* (Kihara et al., 2014), were identified and further investigated. In addition to DIVs found in agriculturally relevant species such as cotton (*Gossypium hirsutum*), Brussel sprouts *(Brassica oleracea*), and sweet potato (*Ipomoea batatas*) (Röse and Tumlinson, 2005; Connor et al., 2007; Meents et al., 2019), more recent studies included traditional medicinal plants and trees (Fontana et al., 2009; Martins et al., 2017; Kanagendran et al., 2018; Portillo-Estrada and Niinemets, 2018). Although the inclusion of a wider array of species highlights common DIV constituents, the potential as a functional DAMP yet remains to be verified for the majority of them. Quintana-Rodriguez and colleagues compiled valuable information regarding VOCs triggering responses at multiple levels, identifying, e.g., GLVs, methanol, and MeJA as resistance-enhancing compounds (Duran-Flores and Heil, 2016; Quintana-Rodriguez et al., 2018). In recent studies, a combination of wounding and additional abiotic stresses (e.g., gasses, temperatures, dark treatments) revealed more volatile profiles in various species (Loreto et al., 2006; Brilli et al., 2011; Kanagendran et al., 2018). However, the focus of these investigations was mainly on the combined stress treatments, just mentioning DIVs for the sake of completeness of individual effects and not for its sole purpose. Apart from studies investigating the physiological and ecological role of DIVs, research unraveling the impact of wounding on plant volatile composition during food processing has also entered the global industry (Moretti et al., 2002; Farneti et al., 2013; Zeng et al., 2016).

## Wounding Versus Wounding – Pitfalls in Standardization

A crucial aspect of all studies implementing artificial wounding is the standardization and reproducibility of such methods, especially regarding the comparability of obtained results. As discussed by Heil, mechanical damage was shown to be sufficient to induce responses in various species that are comparable to those observed after herbivore feeding – however not in all cases (Heil, 2009). The ambiguity of reports containing artificial wounding is mainly caused by the flexibility of the treatment itself. As recently highlighted by Waterman et al. (2019), the execution of mechanical damage can comprise cutting, scratching, piercing, grinding, or pinching of leaf areas differing in size whilst in- or excluding the midrip; therefore resulting in highly variable responses even within the same species (Mithöfer et al., 2005). Regarding its effect on VOC release, artificial wounding is known to produce elevated DIV levels (see above) although not as intense and diverse as during herbivory (Fontana et al., 2009; Holopainen and Gershenzon, 2010). These shortcomings were omitted by adding specific elicitors or oral secretion obtained from the respective herbivore. Furthermore, the construction of a robotic caterpillar (‘MecWorm’) revealed that continuous mechanical damage simulates herbivory more accurately than single wounding events, yielding DIV patterns comparable to actual herbivory (Mithöfer et al., 2005). Taken together, although the possibility of standardized wounding patterns to study DAMPs and DIVs in a comparable manner exists, the extent of reported artificial damage still varies tremendously.

## Which DIVs Can Elicit Downstream Responses on a Molecular Level?

While DAMPs activate defense-related signaling such as membrane depolarization, cytosolic Ca^2+^ concentration changes, generation of reactive oxygen species (ROS), MAPKinase activation, octadecanoid (jasmonate) and/or salicylic acid (SA) signaling, as well as downstream defense responses like the expression of digestion inhibitors and of defense-related genes (Duran-Flores and Heil, 2016; Li et al., 2020), our knowledge of VOC-induced defense-related responses is still fragmentary. In particular studies on early signaling events are missing. To answer the question whether any of the volatiles mentioned above could actually function as a DAMP, either systemically or between plants, it is crucial to consider whether they are (i) emitted after mechanical damage only and (ii) possess the ability to trigger detectable downstream responses on a molecular or physiological level. Although the exact mechanism of volatile perception still remains an enigma, evidence for perception of DIVs in trees, e.g., sugar maple (*Acer saccharum*) and poplar (*Populus x euroamericana*), was already found by Baldwin and Schultz (1983). This study demonstrated that airborne cues emitted from trees with artificially torn leaves triggered an enhanced accumulation of phenolic compounds and condensed tannins in nearby undamaged individuals. Over the following 20 years, an extensive amount of research was published, identifying specific DIVs and their ability to induce a plethora of responses in a broad spectrum of species ranging from trees, shrubs (sagebrush) to crops (cotton, tomato, potato), and model organisms (*A. thaliana*). The main observed responses to DIVs included accumulation of secondary metabolites, especially phenolic compounds and tannins (Baldwin and Schultz, 1983; Zeringue, 1987; Choi et al., 1994), upregulation of proteinase inhibitor gene expression and proteinase inhibitor biosynthesis (Farmer and Ryan, 1990; Reid, 1995), activation of defensive oxidative enzymes (Karban et al., 2000) by compounds such as MeJA or ethylene, which could even lead to an enhanced herbivore resistance (Karban et al., 2003).

A groundbreaking study by Arimura et al. (2000) continued to disentangle the impact of individual compounds in the upregulation of defense-related genes in lima bean. It was demonstrated that only VOCs emitted by *T. urticae-*infested leaves resulted in the upregulation of defense-related genes encoding pathogen-related (PR) proteins including PR-2 (β-1,3-glucanase), PR-3 (chitinase), as well as lipoxygenase (LOX), phenylalanine ammonia-lyase (PAL), and farnesyl pyrophosphate synthetase (FPS), whereas exposure to VOCs from artificially damaged plants only slightly triggered PR-2 gene upregulation. Although VOC emission profiles revealed the presence of (*Z*)-3-hexenol, α-pinene, (*E*)-β-ocimene, DMNT, α-copaene, junipene, β-caryophyllene, and MeSA after artificial wounding by punching holes into the detached leaves, the available concentration of the individual compounds was seemingly not sufficient to trigger defense mechanisms. Follow-up studies with whole plants revealed that GLVs such as (*Z*)-3-hexenol, (*E*)-2-hexenal, and (*Z*)-3-hexenyl acetate were in fact able to induce the expression of defense genes in non-infested plants (Arimura et al., 2001; Farag et al., 2005). Findings by Bate and Rothstein (1998) corroborated the importance of C_6_- GLVs (mainly (*E*)-2-hexenal) triggering plant defense response genes in *A. thaliana.* Additionally, the potential of DIVs such as DMNT or β-ocimene to activate transcript accumulations, if present in high enough amounts, was shown by their individual application resulting in upregulation of several defense genes (Arimura et al., 2000). A similar observation was made by Meents et al. (2019) showing that VOCs released after mechanical wounding with tweezers or the application of DMNT only induced several defense genes and trypsin inhibitors in sweet potatoes in a cultivar- and concentration-specific manner. Both studies highlight the potential of single components as putative DAMPs; however the experimental setup, execution and magnitude of artificial wounding, air exchange, incubation time, and concentration of applied volatiles need to be critically taken into account.

More recent findings focused on the effect of mainly HIPVs in intra- and interspecific plant signaling, omitting artificial treatments and placing VOC signaling in a more ecological context. Matthias Erb and his team found the mainly herbivory-induced aromatic compound indole (Figure 1) to be a potent priming agent in maize (*Zea mays*) which increased the accumulation of defense-related phytohormones and volatiles in undamaged neighboring plants (Erb et al., 2015). Although the indole-mediated priming response was specific for maize only, exposure to synthetic indole triggered the emission of DMNT, α-pinene, and (*E*)-β-caryophyllene also in cotton and cowpea (*Vigna unguiculata*) (Erb et al., 2015). This highlights the potential of indole as a putative universal information transmitter among various species based on the fact that – although in small amounts only – it can be found in other species as well (Zeng et al., 2016; Li et al., 2019; Meents et al., 2019). Again, the mode of damage seems to play a crucial role for defense upregulation, based on studies showing the occurrence of small amounts of indole only after continuous mechanical wounding in certain species (Bricchi et al., 2010; Zeng et al., 2016; Meents et al., 2019) and not after single wounding events (Zhuang et al., 2012). These observations highlight that VOCs mainly declared as HIPVs are not necessarily limited to herbivory, but might also act as a damage-inducible priming agent and triggering DAMP mechanisms with sufficient indole released after wounding. Taken together all of these findings, there is a strong evidence for some DIVs regulating as volatile DAMPs various plant responses *via* different pathways.

How these DAMP signals act on and in neighboring plants and the receiving tissue is still not known. For sure, plants harbor the potential to perceive and transmit volatile signals. Some scientists highlighted the ability of DIVs to further induce VOC emissions in the receiver plant, e.g., via upregulating ethylene biosynthesis genes in lima bean (Arimura et al., 2002), local and systemic terpene production in tomato (Farag and Paré, 2002), or production of HIPVs-mimics in cotton, tobacco (*Nicotiana attenuata*), or maize as a response to MeJA or (*Z*)-3-hexen-1-ol (Halitschke et al., 2000; Rodriguez-Saona et al., 2001; Ruther and Kleier, 2005). Especially airborne MeJA connects different possible pathways, being taken up by the plant and consecutively converted into jasmonic acid and its active conjugates (Tamogami et al., 2008). Jasmonic acid and its conjugates are then able to regulate defense responses including VOC emission; sometimes in cooperation with peptide signaling as shown for prosystemin in tomato (Degenhardt et al., 2010). However, as shown for sweet potato, DIV-induced defense is not necessarily connected with the activation of the jasmonate pathway (Meents et al., 2019). These observations support the possibility of dual functions of certain volatile DAMPs such as DMNT, which could act with and without including known defensive pathways. Moreover, such DAMPs can either directly initiate defense as in the case of sweet potato (Meents et al., 2019) or being involved in priming (Erb et al., 2015).

It should be noticed that DIVs must also be seen in the original sense of tissue damage; i.e., this cue is not necessarily exclusively triggered in the event of an herbivore or pathogen attack but might be involved in activation in vital wounding repair mechanisms within the damaged individual, therefore serving as a shortcut. However, to our knowledge, volatile DAMPs-related to wound healing processes in plants have not been described yet.

## Specificity, Stability, and Range of Influence

One recurring point of controversy has been the distance over which HIPV signals can be received by plants (Baldwin et al., 2002; Karban et al., 2003; Kessler et al., 2006). Recent work has shown that vascular constraints on systemic induction can be overcome with HIPVs (Karban et al., 2006; Frost et al., 2007; Heil and Bueno, 2007), as hypothesized by Farmer (2001) and Orians (2005). However, the potential of emitted VOCs to trigger a systemic response in the emitter or conspecific individuals is a complex interplay of various factors starting from released concentrations of active compound, cue specificity, stereochemistry-related configuration, field *vs* laboratory conditions, and the distance to the emitter (Figure 3) (Preston et al., 2004).

Among several well-studied volatiles, MeJA gained increasing attention from the 90s on after a study by Farmer and Ryan (1990) finding its emission significantly increased after excision of branches from sagebrush. Being conducted in enclosed bell jars only, Karban and colleagues transferred this knowledge to the field, performing further experiments demonstrating that wild tobacco plants growing near clipped sagebrush exhibit less herbivore damage than individuals without a wounded neighbor present (Karban et al., 2000), highlighting the defensive ability of DIVs. Upon further characterization of the emitted plume after mechanical damage in sagebrush, Preston et al. (2004) identified *cis-*MeJA as the main released epimer compared to the *trans* conformation. Subsequent experiments aiming to reproduce the emission of MeJA via application of lanolin paste or aqueous sprays revealed that neither *cis*- nor *trans*-MeJA elicited direct defenses in *N. attenuata* when applied in concentrations consistent with sagebrush emissions. This study exquisitely addressed that besides structural specificity, the application and the released amount of compounds is a crucial aspect making it tremendously difficult to treat plants in physiologically relevant quantities in order to reproduce observations made in the field.

Follow-up field studies on sagebrush conducted by Karban et al. (2006) found air contact and proximity of conspecific plants to be key to intra- and interplant communication. It was shown that adjacent conspecifics of clipped sagebrush were not only influenced within a range of 15 cm but even up to 60 cm. Additionally, a downwind airflow toward the neighboring plant was necessary to establish volatile-mediated contact, ultimately triggering induced resistance among branches as well as within the wounded individual itself which was not observed by clipping and trapping released DIVs (Karban et al., 2000, 2006). In the case of neighboring tobacco plants, 5 days of exposure to clipped sagebrush increased the overall resistance for the whole season with up to 48% decreased herbivore damage (Karban, 2001; Karban et al., 2003). All of these findings underlined the possible longevity of volatile-based protective mechanisms even across different species, however suffering limitations based on airflow and spatial distribution of such cues. Considering the proximity of neighboring individuals, MeJA-based communication appears to be useful in sagebrush due to adjacent plants growing within a maximum distance of 60 cm apart (Karban et al., 2006) including the branches of the clipped individual itself.

Apart from warning neighboring (potentially eavesdropping) individuals, DIVs might also provide a fast and efficient mechanism of within-plant-signaling, reaching further locations of the wounded plant itself as has been demonstrated in lima bean, poplar, blueberry (*Vaccinium corymbosum*), and sagebrush (Karban et al., 2006; Frost et al., 2007; Heil and Bueno, 2007; Rodriguez-Saona et al., 2009; Heil and Adame-Álvarez, 2010). Depending on the growth form, Heil and Karban predicted that large and anatomically complex plants (especially lianas and vines) are more prone to use VOC-mediated protective mechanisms, omitting a time-consuming signaling cascade via the vascular system (Heil and Karban, 2010). Evidence for this hypothesis and the distance over which VOCs can travel was found in lima bean plants grown in the field. Heil and Adame-Álvarez (2010) demonstrated that cues from emitter plants triggered with JA or benzothiadiazole (BTH) increased secretion of extrafloral nectar as an output for resistance in independent receiver plants at a distance up to 50 cm. Interestingly, over 80% of the leaves located around a single leaf at this range still belonged to the same plant, therefore inducing resistance mainly in the same individual (Figure 3) (Heil and Adame-Álvarez, 2010). Additional findings were presented by Girón-Calva et al. (2012) highlighting the specificity of plant perception in lima bean, depending on the applied VOC and the dose and exposure time. Taken together, volatiles are representing a cue for within-plant-signaling as well as an alarm signal for surrounding plants of a possible threat, however in a limited range from 15 up to 60 cm, which was extended to 100 cm by work of Piesik et al. (2010) for some cereal crops and recently by Sukegawa et al. (2018) in a mint (*Mentha × piperita*) emitter – soybean (*Glycine max*) receiver system.

## How Atmospheric Effects Can Shape Volatile Distribution Patterns

In nature, plants are exposed to a vast number of environmental stimuli and stress factors, leading to drastic physio-chemical changes in the plant. These external factors are often omitted in studies that are performed in the laboratory. As addressed in a review by Holopainen and Gershenzon (2010), the co-occurrence of biotic and abiotic stresses such as high temperatures, nutrient availability in the soil, and increasing herbivore attacks, can significantly alter the volatile profiles in plants. These effects can be additive and result in an increased VOC emission, as observed in maize and lima bean (Gouinguené and Turlings, 2002; Vuorinen et al., 2004) under high temperature or ozone stress combined with herbivory, or prioritize a single response, e.g., anti-pathogen instead of anti-herbivore defense (Rostás et al., 2006). Strikingly, after degradation or condensation on leaf surfaces VOCs can play an entirely different biological role (Holopainen and Gershenzon, 2010).

As worked out recently, many different physico-chemical parameters can affect the occurrence and concentration of released VOCs in the close environment. Their particular vapor pressure, but also temperature, wind speed, relative humidity, and radiation are such factors (Figure 3) (Douma et al., 2019). In addition, an important key factor for volatile communication is the atmospheric lifetime of emitted VOCs which can range from 30 s up to several days (Atkinson and Arey, 2003). As stated again by Douma and colleagues, the chemical class of a certain compound is less important than its reactivity with atmospheric oxidants, biosynthesis rate, and volatility (Douma et al., 2019). Thus, the longevity of such a signal strongly depends on the presence of reactive radicals (OH, NO_3_, O_3_) and the number of C double bonds (Mofikoya et al., 2017). Especially ozone, known as the most important tropospheric air pollutant in rural areas (Ashmore, 2005), is highly reactive with a variety of VOCs (Pinto et al., 2007). As demonstrated by Blande et al. (2010) in laboratory studies, this can lead to a significantly decreased signaling distance and, hence, limited plant–plant communication. In numbers, the exposure of *T. urticae*-infested lima beans to 80 ppb ozone (representing concentrations of semi-urban areas) reduced VOC signaling distances from 70 cm (control) to 20 cm, mainly due to degradation of compounds such as (*E*)−β−ocimene, DMNT, and TMTT. Additionally, recent field studies revealed that priming of cabbage (*Brassica oleracea* var. *capitata*) after exposure to HIPVs of *Pieris brassicae*-infested neighbors was significantly disturbed (Girón-Calva et al., 2017) by elevated tropospheric ozone levels, therefore inhibiting a crucial VOC-mediated protective mechanism of plant communication. However, this adverse effect does not apply to all compounds and plant responses. Compounds such as MeSA or 2-butanone were not significantly affected and exposure to even higher ozone concentrations (160 ppb) stimulated extrafloral nectar production in lima bean, representing an increased defense mechanism (Blande et al., 2010). Apart from its influence in the plant itself, oviposition by *P. xylostella* was generally lower in plots under elevated ozone (Mofikoya et al., 2017), indicating that behavioral patterns by the herbivore are also altered in the process. The question whether this activation of defensive mechanisms might be used as a plant protection strategy or simply puts the plant under constant stress, still remains to be answered. All of these findings create a rather puzzling image regarding the benefit or drawback of air pollutants on plants and their VOCs; however representing a major external factor that has to be considered when applying VOCs in the field.

## Volatile DAMPs – Are They Useful in Agriculture?

Over the last decades, numerous studies proposed the use of plant-based VOCs (DIVs as well as HIPVs) for crop protection as means for an environment-friendly pest management (Table 1). All having the same aim, various strategies have been suggested targeting different volatile-based mechanisms. Groundbreaking field studies by Pickett and colleagues (Cook et al., 2007; Hassanali et al., 2008; Pickett et al., 2014) introduced the push-pull-system by intercropping repellant and attractant plant species, luring pests toward attractive odors whilst protecting the important crop from damage.

**TABLE 1:** Overview of plant-derived DIVs and their application in the field.

Compound/molecule class	Classification	Emitter/source	Receiver plant	Applied VOC dosage	Distance emitter-receiver	Response	References (and ref. therein)
**Methyl jasmonate (MeJA)**	DIV	*Artemisia tridentata* (clipped)	*Nicotiana attenuata*	20–30 ng/g FW/h	15 cm	↑Polyphenol oxidase ↑Herbivore resistance	Karban et al., 2000
**Methyl jasmonate (MeJA)**	DIV	*Artemisia tridentata* (clipped)	*Artemisia tridentata*	n.a.	0–60 cm	↑Herbivore resistance	Karban et al., 2006
**Methyl jasmonate (MeJA)**	DIV	Dispenser (Chem-Tica sachet)	*Vitis labrusca* (var. Concord)	1 g; 7 mg/d released	0–30 m	↑Parasitoid abundance	James and Grasswitz, 2005
**Methyl salicylate (MeSA)**	DIV	MeSA dispenser (Predalure)	*Fragaria × ananassa*	2 g/lure	0–10 m	→Pest abundance	Lee, 2010
**Methyl salicylate (MeSA)**	DIV	Dispenser (Chem-Tica sachet)	*Vitis labrusca* (var. Concord)	5 g; 40 mg/d released	0–30 m	↑Parasitoid abundance	James and Grasswitz, 2005
**Methyl salicylate (MeSA)**	DIV	Dispenser (Chem-Tica sachet)	*Vitis labrusca* (var. Concord)	5 g; 60 mg/d released	0–30 m	↑Parasitoid abundance	James and Price, 2004
**Methyl salicylate (MeSA)**	DIV	Dispenser (Chem-Tica sachet)	*Humulus lupulus*	5 g; 60 mg/d released	0–30 m	↑Parasitoid abundance	James and Price, 2004
**(*Z*)-3-Hexenyl acetate**	DIV	Dispenser (Chem-Tica sachet)	*Vitis labrusca* (var. Concord)	1 g; 7 mg/d released	0–30 m	↑Parasitoid abundance	James and Grasswitz, 2005
**(*Z*)-3-Hexenyl acetate**	DIV	Lanolin paste	*Phaseolus lunatus*	30 ng/μl; 10 ng/h released	1 m	↑Height and biomass ↑Flower and fruit production ↓Herbivore damage ↓Cyanide production	Freundlich and Frost, 2019
**(*Z*)-3-Hexenyl acetate**	DIV	Lanolin paste	*Capsicum annuum* (var. Cayenne)	30 ng/μl; 10 ng/h released	1 m	↓Height and biomass ↓Flower and fruit production →Herbivore damage	Freundlich and Frost, 2019
**(*E*)-β-Caryophyllene**	DIV/HIPV*	*Zea mays* ssp. *parviglumis*	n.a.	n.a.	1 m	↑Parasitoid abundance	Rasmann et al., 2005
**n.a.**	DIV/HIPV°	*Mangifera indica* (var. Criollo)	n.a.	n.a.	20 cm	↑Parasitoid abundance	Carrasco et al., 2005
**VOC mixture** monoterpenes, GLVs, terpenes, N- and S- containing VOCs, DMNT, (*Z*)-3-hexenyl acetate, (*E*)-β-ocimene	DIV/HIPV	*Brassica oleracea* (var. Capitata)	*Brassica oleracea* (var. Capitata)	n.a.	30 cm	↑VOC emission (priming)	Girón-Calva et al., 2017
**VOC mixture** (*E*)-2-hexenal, (*Z*)-3-hexen-1-yl acetate, (*E*)-β-ocimene	DIV	*Solidago altissima* (cut)	*Glycine max* (cv. Hyokei Kuro-3)	500 mg cut *S. altissima* pieces	0–15 m	↓Leaf damage ↓*Spodoptera litura* damage	Shiojiri et al., 2017
**GLV mixture** (*Z*)-3-hexenal, (*E*)-2-hexenal, (*Z*)-3-hexenyl acetate	DIV	Dispenser	*Zea mays* (var. Tuxpeño Sequía)	0.2 ml	<0.1–1 m	↑Sesquiterpene emission ↑Herbivore damage ↑Herbivore abundance →Parasitism rate	von Mérey et al., 2011
**(*E*)-β-farnesene**	HIPV/cVOC (GMO)	*Triticum aestivum* (cv. Cadenza)	n.a.	Maximum 30.7 μg/plant/h released	0.5 m	→Grain yield →Aphid abundance →Parasitoid abundance	Bruce et al., 2015
**VOC mixture** 1,8-cineole, menthone, menthol	cVOC	*Mentha* × *piperita* (cv. Candy)	*Glycine max* (cv. Tanba−Kuro) *Brassica rapa Phaseolus vulgaris* (cv. Nagauzuramame)	n.a.	50–100 cm	↓Herbivore damage ↑Defense genes	Sukegawa et al., 2018
**Push-pull-intercropping systems**	DIV/cVOC	For a full review see					Pickett et al., 2014
**Plant extracts**	DAMP	For a full review see					Quintana-Rodriguez et al., 2018

DIV, damage-induced volatiles; HIPV, herbivore-induced volatiles; cVOC, constitutive volatiles; GLV, green leaf volatiles; GMO, genetically modified organism; n.a., not applicable. ↓ decrease; ↑ increase; → no change. *VOC emission induced by herbivore feeding. °Beneficial effect observed after herbivory only.

Following up, various publications aimed to identify suitable crop species and cultivars based on their natural ability to release and induce VOC-mediated defenses in adjacent plants. Studies by Piesik et al. (2010) investigated the influence of mechanical damage and herbivory on the VOC emission in common cereals, e.g., wheat, barley (*Hordeum vulgare*), and oat (*Avena sativa*), revealing tremendous differences in quantities of especially GLVs emitted by injured plants. These species-specific differences in DIV quantity could even be observed in different cultivars of the same species in sweet potato (Meents et al., 2019). In both cases, herbivory resulted in the emission of higher amounts and more different VOCs compared to mechanical injury. However, low amounts of released DIVs after mechanical damage were already sufficient to induce the release of GLVs in uninjured crop plants within 1 m distance (Figure 3) (Piesik et al., 2010). The ability of DIVs to trigger an upregulated VOC release in adjacent plants might serve as an interesting starting point of signal amplification within an agricultural land plot. Supposing that artificial wounding of few individuals can trigger upregulation of VOCs in uninjured neighbors, which subsequently serve as relays amplifying the signal, it would be intriguing to test whether it could actually prime or induce resistance in larger areas of one plot. However, the feasibility of this concept strongly depends on the intensity and frequency of the given stimulus, stability and complexity of the signal, the ability of the receivers to perceive and respond to the given stimulus, the longevity of the response, and whether there is a trade-off between defense and yield. At this point, it might be worth to mention a very recent study demonstrating that among released VOCs – GLVs in particular – were the best candidates to indicate herbivore occurrence, suggesting their longer presence in the environment compared with other VOCs (Douma et al., 2019).

Independent of initial stimuli or wounding events, studies by Sukegawa et al. (2018) suggested mint species due to their constitutive emission of resistance-enhancing volatiles as suitable companion plants for soybean, *Brassica rapa*, and kidney bean (*Phaseolus vulgaris*). Cultivation or pre-incubation for up to 7 days in the greenhouse next to mint plants resulted in lowered herbivore damage and transcript accumulation of defense marker genes for up to 8 days. These promising findings confirmed previous studies in potato by Vucetic et al. (2014) highlighting the potential of constitutively emitted aromatic VOCs to elicit defense or priming in crop species. Another field study showed convincingly that repeated weeding-induced release of DIVs from goldenrod (*Solidago altissima*) plants reduced both leaf and seed damage in soybeans. It could be further shown that at least three different goldenrod-derived monoterpenes were involved in the induction of the respective soybean defense (Shiojiri et al., 2017). However as critically pointed out by Sukegawa et al. (2018), one has to consider whether the recipient crop species (such as soybean) is grown in large monocultures in the field, which might drastically attenuate the beneficial effect of mint as companion plants, making it more suitable for small scale house gardening and glasshouse cultivation.

Another interesting principle regarding volatile-based protection comprises the addition of a third trophic level. Various studies (Dicke et al., 1990, 2003; Turlings et al., 1990; Takabayashi and Dicke, 1996; Arimura et al., 2009; Baldwin, 2010) revealed that plants release distinct volatile blends upon herbivory in order to attract natural enemies of the attacking herbivore. Making direct use of this knowledge, researchers tested common HIPVs such as DMNT or (*Z)*-3-hexenyl acetate among many others, in field studies regarding their attractiveness toward parasitoids. In the process, MeSA as both a DIV and an HIPV, was revealed to be a promising candidate for commercial application due to its luring ability of predatory mites, bugs, and lacewings whilst repelling aphid plant pests (Dicke and Sabelis, 1987; Dicke et al., 1990; Drukker et al., 2000; Ozawa et al., 2000; James, 2003, 2005). Although being able to bait certain insect species in hop yards over a distance of 15 m away from the dispenser, studies using commercially available MeSA lures in strawberry (*Fragaria × ananassa*) fields did not result in decreased local pest abundance (Lee, 2010). This study just posing as an example, it nevertheless reveals the complexity of this strategy due to the predator’s preferences and the potential lack of a rewarding system.

Combining aforementioned strategies, studies by von Mérey et al. (2011) constructed dispensers in maize fields releasing synthetic GLVs in order to induce and/or prime defense in neighboring plants while simultaneously monitoring predator and herbivore attractiveness. Although GLV-exposed maize plants emitted increased concentrations of sesquiterpenes, the hypothesis this would improve herbivore resistance could not be maintained but caused even higher numbers of herbivores, depending on the distance to the dispenser. Another crucial aspect is again the emitted concentration of each compound especially in complex mixtures, since repellent cues can be turned into attractants in the process or covering the desired function, especially when presented in the wrong context (D’Alessandro and Turlings, 2005; Mumm and Hilker, 2005; Snoeren et al., 2010). As addressed by Heil and Walters (2009) (VOC-mediated) induced systemic resistance seems to come with ecological costs. This effect is again highly species-specific and strongly dependent on the applied volatile, which was shown in a field study where lima bean and pepper (*Capsicum annuum*) were exposed to low doses of (*Z*)-3-hexenyl acetate for 7 days (Freundlich and Frost, 2019). Volatile treatment resulted in increased leaf and flower formation, overall taller growth and decreased herbivory in lima bean plants, however coming at the cost of a reduced cyanide induction (trade-off). An entirely different output was observed in pepper, producing fewer flowers and fruits conjoined with reduced above- and belowground biomass and unaltered herbivore damage. These observations illustrate the effect of a single VOC on traits such as reproductive fitness and growth in a species-specific manner which very carefully needs to be considered while choosing a suitable VOC-plant pairing in agriculture. Having a large scale application of volatile treatments in agriculture in mind, in addition to the compounds’ environmental compatibility and efficacy also their production costs must be considered, which may become a limiting factor.

## Conclusion

Within the past decades, plant-based signaling compounds became increasingly popular as eco-friendly priming compounds or resistance boosters in the fields of biotechnology and agriculture. Unfortunately, up to now most of the proposed concepts have not yet proven to be successful enough to pose as viable alternatives for conventional crop protection strategies. This observation is mainly based on the variety of drawbacks addressed by Brilli et al. (2019) which still need to be further discussed and overcome in the future. However, new concepts exploring the potential of DAMPs as plant protective compounds found especially eDNA (Ferrusquía-Jiménez et al., 2020) to be a new candidate for application in the field. In addition to such treatments directly spraying compounds produced by wounded plant tissues on unwounded crops, we would like to focus onto damage-induced volatile compounds (DIVs). These DIVs are (i) specifically synthesized and emitted upon tissue disruption and (ii) can serve as intra- and interplant signals initiating immune responses as well. Due to their generation upon injuries or damage, these compounds can also be classified as DAMPs. Mainly GLVs but also DMNT and indole fulfill the criteria to be classified as volatile DAMPs in *stricto sensu*. Their airborne nature opens new possibilities for applications but also reveals new challenges. A general issue is the volatile-based communication itself, involving the plant as an emitter as well as a receiver. On the one hand, even in conspecific plants a high genetic identity does not guarantee a functioning communication between varieties as shown for sweet potato (Meents et al., 2019). On the other, VOC-emitting plants do not necessarily release “private messages” and may attract unwanted organisms as well as advantage eavesdropping adjacent plants competing for nutrients (Gershenzon, 2007). The intensity and longevity of the volatile “messages” itself is highly fluctuating as well since environmental conditions can strongly reduce the efficiency of the particular volatile compound not only on a physico-chemical level but simply by fast dilution due to strong winds. On a physiological scale, the cost-benefit ratio for the emitting plant and the effect on conspecific individuals need to be further investigated to prove an actual profit and not simply a trade-off. Taken together, up to this point DIVs pose as a promising approach for DAMP-based crop protection – however, mainly restricted to a controlled and space-limited area such as phytochambers and greenhouses.

## Author Contributions

Both authors listed have made a substantial, direct and intellectual contribution to the work, and approved it for publication.

## Conflict of Interest

The authors declare that the research was conducted in the absence of any commercial or financial relationships that could be construed as a potential conflict of interest.
